# Epidural Abscess Following Epidural Evacuation in a Patient With Ventriculoperitoneal Shunt: A Case Report

**DOI:** 10.7759/cureus.19134

**Published:** 2021-10-29

**Authors:** Afaf Shaabi, Rana H Moshref

**Affiliations:** 1 Neurological Surgery, King Fahad General Hospital, Jeddah, SAU

**Keywords:** pediatric, epidural hematoma, infection, craniotomy, hematopoiesis

## Abstract

Epidural abscess is considered one of the most common intracranial infections. We report a pediatric patient with chronic hematopoiesis and thick double periosteal layers who developed an epidural pus collection after epidural hematoma evacuation. This article highlights the importance of detecting complications from epidural hematoma evacuation, including intracranial abscess and pus formation. Therefore, it is crucial to treat such cases meticulously.

## Introduction

Normally, there is a tight connection between the skull bone and the dura, but with increased intracranial pressure, such as tumors, inflammation, and infection, the layers are displaced [1]. The most common intracranial infectious lesions are subdural empyema and epidural abscess, which are characterized by pus sheathed in a capsule [2]. Spontaneous epidural abscesses are rare, accounting for 0.2-1.2 cases per 10,000 hospital admissions per year [3]. Several mechanisms of spread have been postulated, including poor aseptic method, contiguous spread, such as sinusitis, otitis media, postoperative, posttraumatic [4], and systemic routes by a foreign catheter or needle insertion [5]. The main presentation is slight fever, headache with a Glasgow Coma Scale (GCS) 12-15, and a sequel of seizures, hydrocephalus, and infection [6]. Swelling and discharge of pus are noticed following cranioplasty and ventriculoperitoneal shunt (VPS) insertion [7]. The most common organisms are *Streptococcus *(60%), *Staphylococcus aureus*, Gram-negative bacilli, and fungi [1]. Laboratory investigations show leukocytosis in nearly 70% of patients with an erythrocyte sedimentation rate of more than 30 mm/h, and thrombocytopenia indicates a worse prognosis in sepsis [3].

In a retrospective study of 21 patients, provisional antibiotics utilized were amikacin, ceftriaxone, and metronidazole [2]. Then the authors switched to culture-sensitive antibiotics. A decision on surgery or further management was dictated by follow-up computed tomography (CT) imaging 14 days postoperatively and poor neurological outcome [2]. Brain magnetic resonance imaging (MRI) with diffusion-weighted or tensor imaging, perfusion-weighted imaging is more sensitive than CT for brain abscess, particularly in the early stages, but CT remains more widely available and can adequately identify potential abscesses and confirm response to treatment [8,9]. The outcome improved with prompt diagnosis by modern neuroimaging, which led to a decrease in mortality and morbidity of 10-16% with the timely introduction of antibiotics [2,3]. In posterior fossa, multiloculated, or superficial well-circumscribed abscesses, surgical interventions can be used [8].

We report a case of a patient with chronic hematopoiesis with thick double periosteal layers with delayed development of epidural pus collection after one year following epidural hematoma (EDH) evacuation and two years following VPS insertion. There are few examples of extramedullary hematopoiesis recorded in the literature, with less than ten known cases of myeloid metaplasia, polycythemia vera, and sickle cell anemia generating bilateral subdural or epidural hematomas needing evacuation [10].

## Case presentation

Medical history

A 15-year-old female with a known case of epilepsy from four years of age on carbamazepine 400 mg twice daily, communicating hydrocephalus on the right VPS (unknown valve as the surgery was done in an outside hospital), was referred as a lifesaving case of epidural hematoma after falling on a hard playground floor. There was no history of fits, vomiting, or loss of consciousness.

Clinical findings

**Figure 1:**
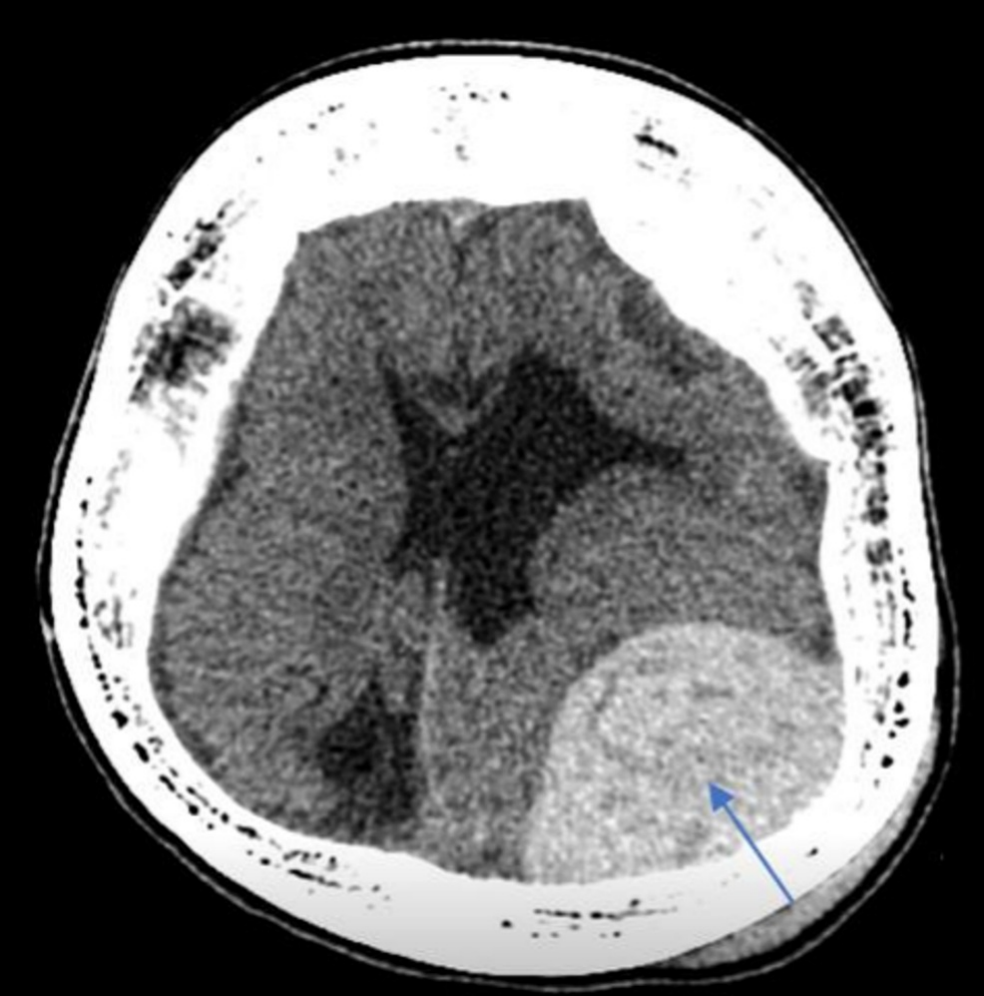
The plain CT brain showed acute left parietal epidural hematoma at the convexity of the left parietal bone (pointed in the image), measuring 9.7 x 5.2 x 8.4 cm in maximum AP, CC, and transverse dimensions with significant mass effect and a midline shift of 4 mm. AP: anterior-posterior, CC: craniocaudal

On examination, she had a subgaleal swelling, no open scalp wounds. On admission, she was vitally stable and afebrile; GCS was 12/15 E3V5M4. Her pupils were 3 mm, equal and reactive. She had right-sided hemiparesis 4/5. The plain CT scan of the brain showed an acute left parietal epidural hematoma at the convexity of the left parietal bone measuring 9.7 x 5.2 x 8.4 cm in maximum anterior-posterior (AP), craniocaudal (CC), and transverse dimensions with significant mass effect (Figure 1).

Timeline and therapeutic intervention

Emergency epidural hematoma evacuation was performed in the first admission. A follow-up CT brain showed an interval decrease in the size of the left parietal epidural hematoma.

**Figure 2:**
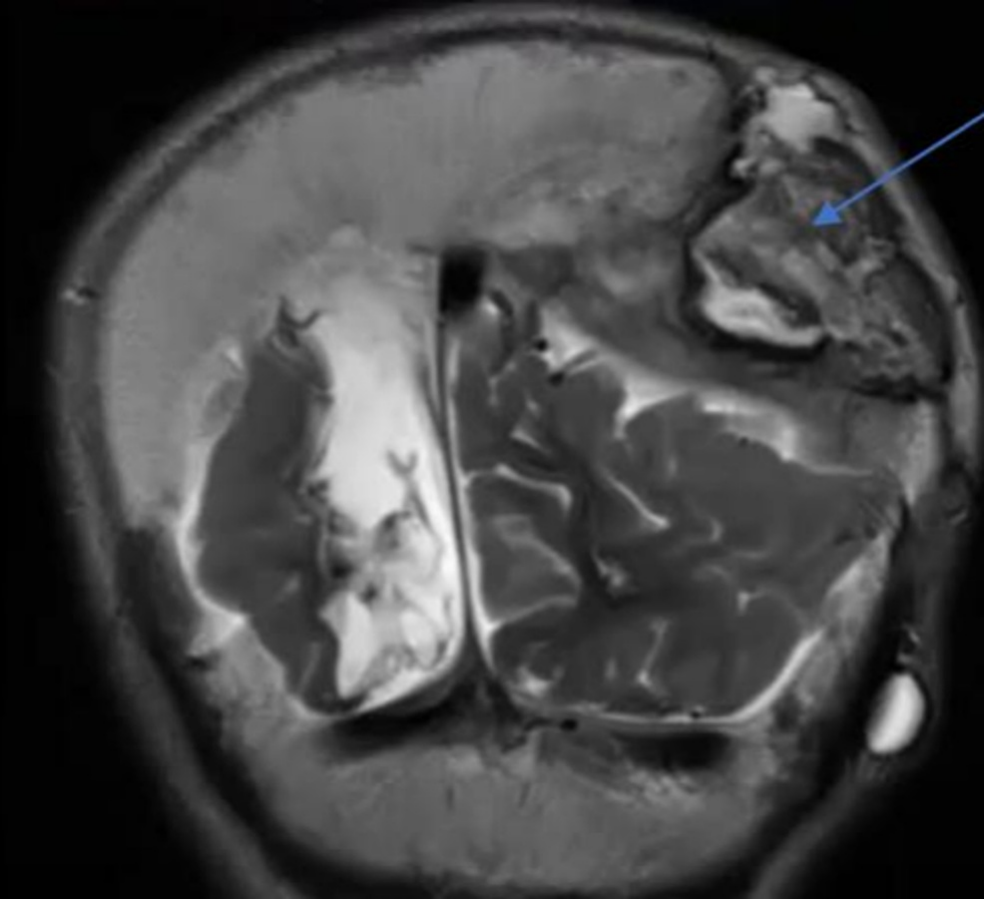
An MRI brain scan showed an enhancing epidural collection of 4 cm thickness (pointed in the image).

She was readmitted one year later with pus discharge from the craniotomy site. She had leukocytosis, with a WBC of 4.5 x 10^9^/L (normal value: 4-10 x 10^9^/L), a high C-reactive protein (CRP) of 72.2 mg/L (normal value: 0-3 mg/L), and an erythrocyte sedimentation rate (ESR) of 24 mm/h, (normal value: 0-3 mm/h). The craniotomy site in the left parietooccipital region was fistulized and had yellowish offensive pus discharge. The culture showed mixed flora, and she was started on cefazolin. An MRI brain scan showed a rim-enhancing epidural collection of 4 cm thickness with dural enhancement and a superficial collection measuring about 4 x 1.4 cm (Figure 2). She underwent epidural pus evacuation, where the scar was incised. There was noticeable pus coming from the skull bone flap from the epidural space, which was evacuated till the dura was exposed and was sent for analysis. Curettage of bone edges was done, and good hemostasis was achieved to avoid removal of the bone. The outer skull bone table was fixed with miniplates and screws. The closure was done using Prolene. Pus culture was taken preoperatively and showed *Staphylococcus aureus*, and the patient was started on vancomycin 300 mg every six hours intravenous route (IV) for two weeks. Shunt tapping was done to make sure there was no underlying ventriculitis, and cerebrospinal fluid (CSF) culture was negative.

**Figure 3:**
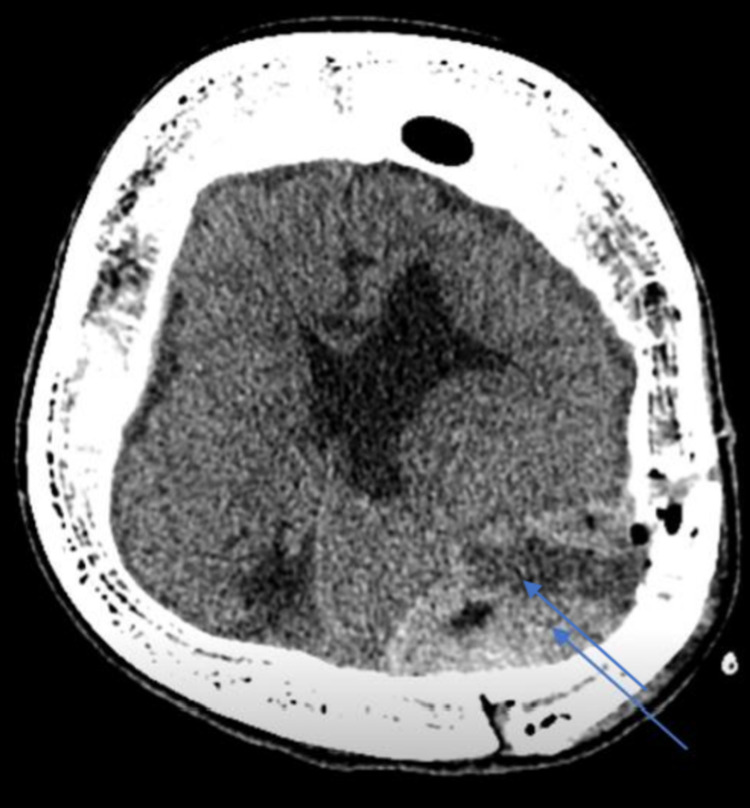
CT brain done three weeks postoperatively showed superficial collection beneath the surgical defect, extra-axial collection with an irregular enhancing rim measuring approximately 4.2 x 4 cm (maximum axial dimension) (pointed in the image).

**Figure 4:**
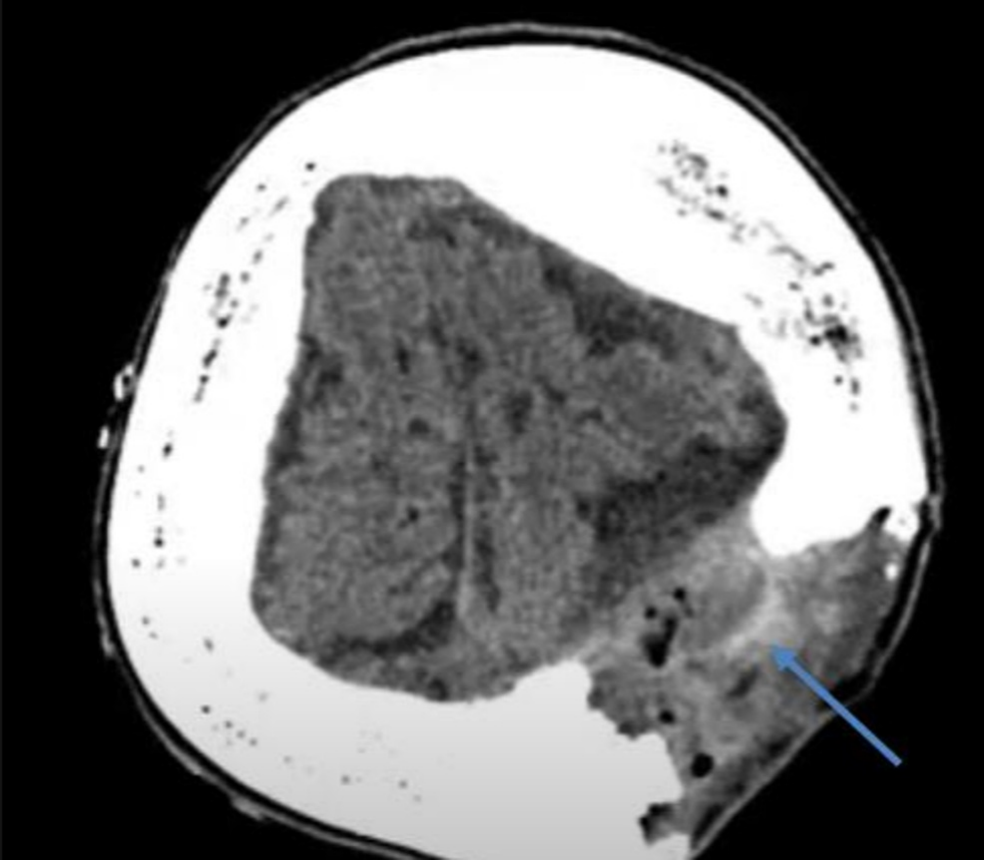
CT brain done showed redemonstration of operative bed subgaleal and epidural heterogenous marginally enhancing collection measuring 7 x 4.5 x 4.5 cm (pointed in the image).

A CT brain was done three weeks postoperatively as the patient was still complaining of low-grade fever. The CT brain showed superficial collection beneath the surgical defect, extra-axial collection with an irregular enhancing rim measuring approximately 4.2 x 4 cm (maximum axial dimension) (Figure 3). The patient underwent pus evacuation and infected bone flap removal. An old craniotomy flap incision was reopened with debridement of infected skin edges and removal of infected subgaleal necrotic pus tissue. The infected bone flap was removed and discarded, as well as the epidural pus collection and all infected granulation tissue. Then the site was irrigated with saline, betadine, and gentamycin. Further nibbling of the bone edges was done till the healing bone was reached. The closure was done in a single layer of interrupted mattress stitches. Wound culture showed pansensitive *Staphylococcus aureus,* and she was on ciprofloxacin 250 g twice daily orally and amoxicillin-clavulanic acid 625 mg thrice daily orally for a course of four weeks. The last CT brain done four weeks after the course of antibiotic administration showed redemonstration of the operative bed subgaleal and epidural heterogenous marginally enhancing collection measuring 7 x 4.5 x 4.5 cm (Figure 4). CRP and ESR returned to normal. She was afebrile, and the wound was clean; she was ready for discharge on an amoxicillin-clavulanic acid 625 mg thrice daily two-week course.

Follow-up and outcome

A six-month follow-up revealed that the infection had not returned clinically and radiologically.

## Discussion

We describe a patient who developed perioperative complications of epidural hematoma (EDH) evacuation and then underwent epidural pus evacuation after one year from EDH evacuation. According to the literature, there has been a postulated reason for collection, such as itching, which can cause wound dehiscence, such as in this patient [5]. The technical difficulties include reoperation and recollection of epidural pus. The patient had double layers of thick periosteum, which led to increased pus collection in a contained compartment with minimal clinical findings. The decision to keep the bone was made to reserve the bone as the patient of pediatric age group, and there was no gross spillage intracranially, but this decision led to the infected bone. In previous studies, it has been advocated to remove the infected bone along with the evacuation of the pus collection [8].

## Conclusions

This article is written to report possible complications from the epidural hematoma evacuation, including intracranial abscess. Therefore, it is crucial to treat such cases meticulously with antibiotics, debridement, and removal of the infected bone to prevent a recurrence. Follow-up with inflammatory markers and a CT brain scan are required to determine treatment success. 
